# No Detectable Spatial Cognitive Impairment Despite Acute Sensorimotor Deficits Following Transient Middle Cerebral Artery Occlusion in Rats: A Post Hoc Exploratory Analysis

**DOI:** 10.1002/brb3.71588

**Published:** 2026-07-09

**Authors:** Michael Veldeman, Mark Coburn, Anke Hoellig

**Affiliations:** ^1^ Department of Neurosurgery University Hospital RWTH Aachen Aachen Germany; ^2^ Department of Anesthesiology University Hospital RWTH Aachen Aachen Germany; ^3^ Department of Anesthesiology and Intensive Care Medicine University Hospital Bonn University Bonn Bonn Germany

**Keywords:** cerebral ischemia, rat, stroke, transient middle cerebral artery occlusion

## Abstract

**Introduction:**

Ischemic stroke is a leading cause of mortality and long‐term disability, and many survivors develop persistent cognitive deficits that severely affect quality of life. Transient intraluminal filament MCAO models are widely used to study stroke pathophysiology and recovery, yet reported cognitive outcomes are inconsistent. Apparent impairments on spatial tasks such as the Morris water maze (MWM) may reflect residual sensorimotor limitations rather than true mnemonic dysfunction. Here, we performed a post hoc, longitudinal analysis of behavioral data from a 2‐h tMCAO cohort to determine whether this model produces measurable deficits in spatial learning, memory, or cognitive flexibility.

**Material and Methods:**

Male Wistar rats underwent Sham surgery or 2‐h tMCAO using the intraluminal filament technique with laser Doppler confirmation of adequate occlusion (> 60% cerebral blood flow reduction) and reperfusion. Predefined quality criteria led to exclusion of animals with insufficient occlusion/reperfusion or hemorrhage, yielding final groups of Sham (*n* = 14) and tMCAO (*n* = 10). Behavioral testing was conducted by masked assessors across 30 days and included neurological scoring (days 1, 7, and 30), MWM acquisition (days 3–7) with probe testing (Day 8), reversal learning (days 24–28) with probe testing (Day 29), and open field assessments (days 3, 4, 5, and 29). Repeated‐measures outcomes were analyzed using linear mixed‐effects models.

**Results:**

tMCAO animals exhibited marked acute sensorimotor deficits on Day 1 that improved by Day 7 and largely resolved by Day 30. MWM performance showed intact spatial acquisition, preserved spatial memory on Day 8, and unimpaired reversal learning. Open field testing revealed no locomotor, exploratory, or anxiety‐like deficits.

**Conclusion:**

Within the testing window of this post hoc analysis (days 3–30), 2‐h tMCAO in male Wistar rats produced transient sensorimotor impairment but no detectable spatial cognitive deficits. This exploratory finding raises the possibility that, under stringent quality control, this model may be less well‐suited for studying post‐stroke cognitive impairment, suggesting consideration of sensorimotor endpoints or alternative paradigms when cognitive dysfunction is the primary target. As a post hoc analysis, this study additionally exemplifies the principle of reduction within the 3Rs framework, deriving scientific value from an existing dataset without additional animals.

## Graphical abstract

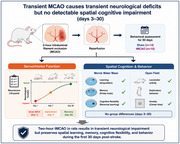

**Abbreviations**
ARRIVE: Animal Research: Reporting of In Vivo Experiments
AZ: Aktenzeichen (file/reference number for ethical approval)
MCAO: middle cerebral artery occlusion
MWM: Morris water maze
SD: standard deviation
SEM: standard error of the mean
tMCAO: transient middle cerebral artery occlusion

## 1 Introduction

Ischemic stroke represents a major global health burden, ranking among the leading causes of death and long‐term disability worldwide (Donkor 2018; Liu et al. 2025). With population aging in developed nations, the societal and economic impact of stroke continues to escalate, necessitating improved therapeutic strategies and a deeper understanding of functional recovery mechanisms (Wang et al. 2025). Preclinical stroke research relies heavily on rodent models of middle cerebral artery occlusion (MCAO) to investigate pathophysiological mechanisms and evaluate potential therapeutic interventions (Bederson et al. 1986; Shiraishi and Simon 1989). Among these, the intraluminal filament model producing transient MCAO (tMCAO) has achieved widespread adoption owing to its technical reproducibility, adjustable occlusion duration, and capacity to simulate the reperfusion conditions encountered clinically following recanalization therapy (Koizumi 1986; Themistoklis et al. 2022). The neurological consequences of experimental MCAO have been characterized using neurological scoring systems that document acute sensorimotor deficits (Garcia et al. 1995; Wakayama et al. 2007), yet the cognitive sequelae and their longer term time course remain less consistently defined (Li et al. 2013; Gupta et al. 2002). Human stroke patients frequently exhibit cognitive impairment, including memory dysfunction, attentional deficits, executive dysfunction, and spatial disorientation, which persist beyond resolution of motor deficits and substantially impact functional independence and quality of life (El Husseini et al. 2023). Whether rodent MCAO models faithfully recapitulate these cognitive consequences has been debated, with conflicting reports regarding spatial learning and memory deficits in the Morris water maze (MWM) and other cognitive tasks following experimental stroke.

This inconsistency in reported cognitive outcomes may reflect methodological heterogeneity across studies, including variations in occlusion technique (permanent vs. transient), occlusion site (proximal vs. distal), occlusion duration, species and strain differences, assessment timing, and behavioral testing protocols. Critically, interpretation of apparent learning deficits may be confounded by coexisting sensorimotor impairments, motivational alterations, or procedural learning difficulties that artificially inflate measures such as escape latency or path length in water navigation tasks. Bingham et al. (2012) demonstrated that conventional behavioral endpoints may misattribute sensorimotor dysfunction to cognitive impairment, advocating for refined testing protocols incorporating probe trials, training to predetermined performance criteria, to differentiate mnemonic from motor contributions to task performance.

The present study represents a post hoc analysis of behavioral data originally collected as part of a preclinical investigation examining neuroprotective effects of noble gas inhalation following experimental stroke (Liu et al. 2019, 2022). During the 30‐day survival period, a comprehensive behavioral assessment was conducted, including clinical neuroscore evaluation, MWM testing of spatial learning and memory, and open field analysis of locomotor and exploratory behavior. The specific objectives of this post hoc analysis were to characterize temporal recovery patterns across sensorimotor, cognitive, and locomotor domains during the 30‐day post‐stroke period in the untreated control and Sham surgery groups, and to determine whether functional dissociations exist between these domains that might inform the interpretation of behavioral outcomes in preclinical stroke research.

## 2 Materials and Methods

### 2.1 Study Design and Ethical Approval

This investigation employed a randomized controlled design to evaluate the time course of functional recovery in a rat model of transient focal cerebral ischemia. The experimental protocol was approved by the State Office for Nature, Environment, and Consumer Protection (Aktenzeichen [AZ] 84–02.04.2013. A418, Landesamt für Natur, Umwelt und Verbraucherschutz Nordrhein‐Westfalen). All experiments were performed in accordance with the German legislation governing animal studies (Tierschutzgesetz, Tierschutz‐Ver‐ suchstierverordnung) and the Animal Research: Reporting of In Vivo Experiments (ARRIVE) guidelines 2.0 (Percie du Sert et al. 2020).

### 2.2 Animals

Male Wistar rats (weighing 250–390 g at 8–12 weeks of age) were procured from Charles River Laboratories (Sulzfeld, Germany). Following delivery, animals underwent a 1‐week habituation period in the animal facility before experimental procedures commenced. Standard housing included a 12‐h light/dark photoperiod with ad libitum provision of standard rodent diet and tap water. Animals were maintained in groups of up to four in standard Makrolon cages in a specific pathogen‐free environment. Welfare assessments were performed twice daily: morning assessments by trained animal care technicians and afternoon assessments by research personnel, including body weight measurement and general health evaluation.

### 2.3 Randomization and Blinding

Animals were randomly assigned to experimental groups using lot drawing. Each animal received a unique identification number, and behavioral testing was conducted by investigators masked to group assignment.

### 2.4 Surgical Procedure

General anesthesia was induced through intraperitoneal administration of a triple‐agent cocktail comprising midazolam (2 mg/kg; Ratiopharm, Ulm, Germany), medetomidine (0.15 mg/kg; Zoetis, Florham Park, NJ, USA), and fentanyl (0.005 mg/kg; Rotexmedica, Trittau, Germany). Anesthetic depth was sustained via hourly intraperitoneal supplementation with 0.03–0.05 mL of this combination. We did not administer reversal agents during recovery. Orotracheal intubation facilitated mechanical ventilation with a 50:50 nitrogen:oxygen mixture.

Arterial access via tail artery catheterization enabled continuous blood pressure monitoring, and electrocardiographic monitoring captured heart rate continuously throughout the procedure. Core temperature was maintained at 37.0°C–37.5°C using a rectal thermometer coupled to a feedback‐regulated heating platform (Physitemp Instruments, Clifton, NJ, USA).

Transient focal brain ischemia was produced using the intraluminal suture technique as previously described (Liu et al. 2019, 2022; Longa et al. 1989; Ryang et al. 2011). After ischemia induction and extubation, animals were monitored twice daily for overall health and recovery, and they received intramuscular metamizole when needed. Early euthanasia was performed if animals showed significant weight loss, inability to access food or water, or severe neurological impairment.

### 2.5 Behavioral Assessment

Testing occurred during the active light phase. The temporal sequence included clinical neurological assessment on days 1, 7, and 30; spatial navigation training in the MWM spanning days 3–7 (four daily trials); open field exploration on days 3, 4, 5, and 29 (single daily sessions); initial MWM probe test on Day 8; spatial reversal training during days 24–28 (four daily trials); final MWM probe test on Day 29; with terminal procedures on Day 30.

### 2.6 Neurological Function Assessment

We evaluated sensorimotor function using an 18‐point composite instrument (Lenzlinger et al. 2004). Daily evaluations were performed from Day 1 through Day 7, with additional assessment on Day 29.

### 2.7 MWM

Spatial learning and memory were assessed using the MWM in a 150‐cm circular tank with a submerged platform and fixed visual cues. Testing comprised three stages: (Donkor 2018) spatial acquisition (days 3–7, four trials per day) to assess learning of the platform location in the southwest quadrant; (Liu et al. 2025) Transfer Trial 1 (Day 8, single 60‐s probe trial with platform removed) to assess spatial memory consolidation; (Wang et al. 2025) reversal learning (days 24–28, four trials per day) with the platform relocated to the northeast quadrant to assess cognitive flexibility; and (Bederson et al. 1986) Transfer Trial 2 (Day 29, single 60‐s probe trial) to assess memory for the reversed platform location. Swimming behavior (latency, distance, velocity, quadrant time, and platform crossings) was recorded and analyzed using the Any‐Maze tracking system.

### 2.8 Open Field Test

Locomotion, exploration, and anxiety‐like behavior were evaluated in a 90 × 90 cm open field arena divided into central and peripheral zones. Rats were tested on days 3, 4, 5, and 29 post‐surgery for 3 min per session, with behavior recorded via Any‐Maze. Measures included horizontal and vertical activity, zone preference (anxiety index), and general activity parameters; the arena was cleaned between trials to remove olfactory cues.

On Day 30, animals were euthanized under deep anesthesia by exsanguination followed by decapitation.

### 2.9 Infarct Volume Assessment

In a subset of tMCAO animals (*n* = 7 of 10), brains were allocated for histological processing at Day 30. The remaining three brains were homogenized for molecular biological analyses, which is incompatible with the fixation required for volumetric assessment. Brains designated for histology were fixed in 4% paraformaldehyde and sectioned into 2 mm coronal blocks as previously described (Liu et al. 2022). Six coronal sections per animal were embedded in paraffin and stained with hematoxylin‐eosin. Infarct volume was calculated using an indirect method by subtracting the non‐lesioned volume of the ipsilateral hemisphere from that of the contralateral hemisphere, normalized to the contralateral hemisphere volume, and expressed as percentage hemispheric loss. These volumetric data were originally acquired as part of the parent study (Liu et al. 2022).

### 2.10 Statistical Analysis

Repeated‐measures data were analyzed using linear mixed‐effects models with group, time (day/timepoint), and their interaction as fixed effects, and animal as a random intercept to account for within‐subject correlation. This approach accommodates missing data using maximum likelihood estimation. Significant omnibus effects were followed by post hoc pairwise comparisons with appropriate adjustment for multiple testing. Standardized effect sizes were calculated as Cohen's d using pooled standard deviations (SDs) and interpreted according to conventional thresholds. A sensitivity analysis was performed using the pwr.t2n.test function (pwr package, R) to determine the minimum detectable effect size (MDES) at 80% power and *α* = 0.05 for the unequal‐sample two‐group design. Model assumptions were evaluated using residual diagnostics. Data are presented as mean ± SD (in the text) or mean ± standard error of the mean (SEM) (in selected figures, as indicated). All tests were two‐tailed with α level set at 0.05. Statistical analyses were conducted using R version 4.5.2 (R Foundation for Statistical Computing, Vienna, Austria) through RStudio (version 2025.09.2+418, Posit Software, PBC, Boston, MA).

The authors used Claude (Anthropic, claude.ai), a large language model, for language editing and refinement of the manuscript. All scientific content, ideas, hypotheses, and conclusions are solely the work of the authors. The AI tool was not involved in study design, data analysis, or interpretation of results.

## 3 Results

### 3.1 Sample Characteristics and Study Completion

The final analytical cohort comprised 24 animals: Sham (*n* = 14) and tMCAO (*n* = 10). All Sham‐operated animals met inclusion criteria, whereas 56.5% of stroke animals (13/23) were excluded based on predefined quality control criteria for occlusion adequacy (> 60% cerebral blood flow reduction) and successful reperfusion. All animals completing inclusion criteria survived the full 30‐day observation period and completed all scheduled behavioral assessments.

### 3.2 . Infarct Volume

Histological assessment of infarct volume was performed in 7 of 10 tMCAO animals, with the remaining three brains allocated to molecular biological analysis as part of the parent study (Liu et al. 2022). All seven animals with available tissue showed measurable infarction, confirming successful stroke induction across the retained cohort. Mean hemispheric loss was 19.4% (range 5.4%–33.4%) (Liu et al. 2022).

### 3.3 MWM—Spatial Acquisition

**FIGURE 1:**
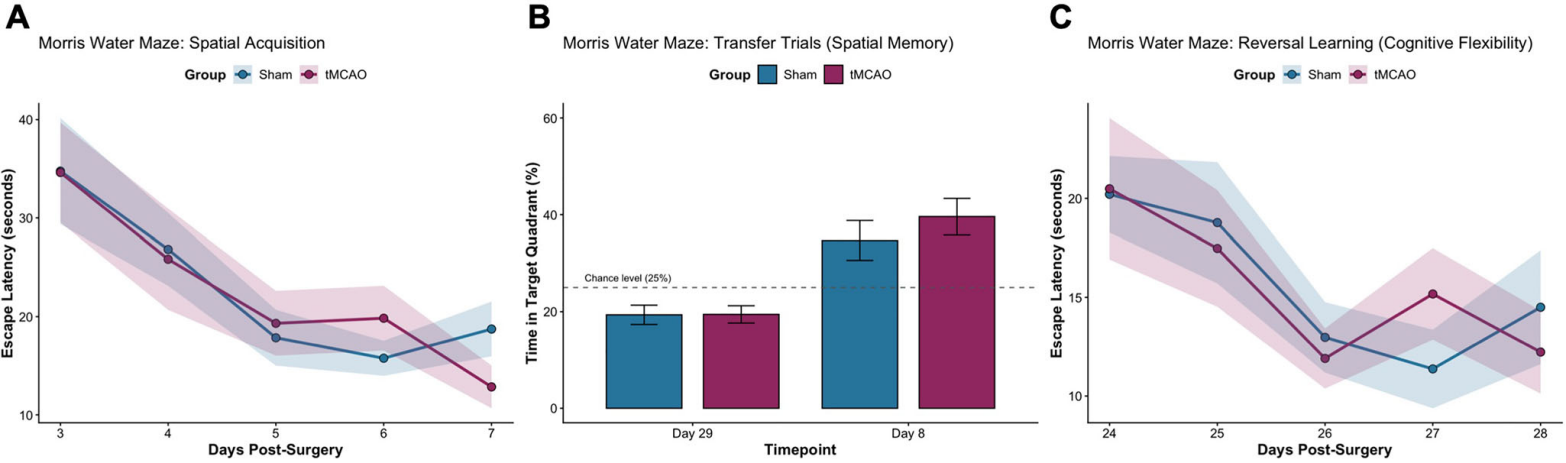
Morris water maze performance following transient middle cerebral artery occlusion. (A) Spatial acquisition. Mean escape latency (± 95% confidence interval) across 5 days of acquisition training (days 3–7 post‐surgery) in Sham (blue, *n* = 14) and tMCAO (magenta, *n* = 10) groups. (B) Spatial memory. Percentage of time spent in the target quadrant during probe trials conducted on Day 8 and Day 29. Dashed horizontal line indicates chance performance (25%). Data presented as mean ± SEM. (C) Cognitive flexibility (reversal learning). Mean escape latency (± 95% confidence interval) during reversal training (days 24–28 post‐surgery) when the platform was relocated to the opposite quadrant. SEM, standard error of the mean; tMCAO, transient middle cerebral artery occlusion.

During the 5‐day spatial acquisition phase (days 3–7 post‐surgery), both experimental groups demonstrated progressive improvement in locating the submerged platform, as evidenced by declining escape latencies across training days (Figure 1A). Linear mixed‐effects analysis revealed a highly significant main effect of Day (*F* (1, 92.2) = 32.86, *p* < 0.001), confirming that animals in both groups successfully acquired the spatial navigation task. Critically, there was no significant main effect of Group (*F* (1, 109.8) = 0.094, *p* = 0.760), indicating that overall learning performance did not differ between tMCAO and Sham animals. The Group × Day interaction was also nonsignificant (*F* (1, 92.2) = 0.137, *p* = 0.712), demonstrating that the rate of learning improvement was equivalent between groups.

### 3.4 MWM—Transfer Trials

Probe trials conducted with the platform removed assessed spatial memory consolidation at two timepoints: Day 8 (24 h post‐acquisition) and Day 29 (following reversal learning). During Transfer Trial 1 on Day 8, both groups demonstrated robust spatial memory, spending substantial time searching the previously reinforced quadrant (Figure 1B). Percentage time in the target quadrant did not differ significantly between groups (Sham: 34.7 ± 15.4% vs. tMCAO: 39.6 ± 12.0%; t (22) = 0.072, p = 0.289). Both groups performed significantly above the chance level of 25% expected from random search (Sham: t (13) = 2.36, p = 0.034; tMCAO: t (9) = 3.63, p = 0.005), indicating successful memory consolidation in both experimental conditions. Secondary measures including annulus crossings over the former platform location showed comparable frequencies between groups (p = 0.458).

Transfer Trial 2 on Day 29 assessed memory for the platform location established during reversal training. Performance in the target quadrant was identical between groups (Sham: 19.4 ± 7.5% vs. tMCAO: 19.4 ± 5.7%; t (22) = −0.019, p = 0.985). Linear mixed‐effects analysis confirmed a significant main effect of Timepoint (F (1, 44.0) = 30.04, p < 0.001), with both groups exhibiting reduced target quadrant preference on Day 29 versus Day 8. However, the Group × Timepoint interaction was not significant (F (1, 44.0) = 0.555, p = 0.460), indicating parallel temporal trajectories of memory retention in both groups.

### 3.5 MWM—Reversal Learning

To assess behavioral adaptability at an extended post‐stroke timepoint, the escape platform was relocated to the diametrically opposite quadrant during days 24–28 (Figure 1C). Both groups successfully learned the new platform location, as evidenced by progressively decreasing escape latencies across the 5‐day reversal period (main effect of Day: *F* (1, 93.2) = 12.40, *p* < 0.001). There was no significant main effect of Group (*F* (1, 94.1) = 0.000, *p* = 0.997), indicating equivalent relearning capacity between tMCAO and Sham animals at this late post‐stroke timepoint. The Group × Day interaction was likewise nonsignificant (*F* (1, 93.2) = 0.000, *p* = 0.999), demonstrating that the trajectory of reversal learning did not differ between experimental conditions.

### 3.6 Open Field Testing

**FIGURE 2:**
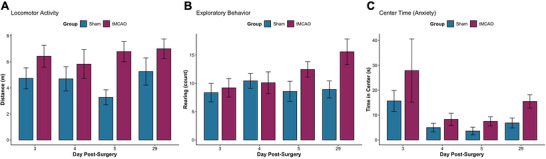
Open field test performance following transient middle cerebral artery occlusion. (A) Locomotor activity. Total distance traveled during 3‐min open field sessions on days 3, 4, 5, and 29 post‐surgery in Sham (blue, *n* = 14) and tMCAO (magenta, *n* = 10) groups. (B) Exploratory behavior. Rearing frequency (vertical exploration) during open field sessions on days 3, 4, 5, and 29 post‐surgery. (C) Center time (anxiety). Time spent in the center zone during open field sessions on days 3, 4, 5, and 29 post‐surgery. Data presented as mean ± SEM. SEM, standard error of the mean; tMCAO, transient middle cerebral artery occlusion.

Open field assessment conducted on days 3, 4, 5, and 29 revealed no evidence of locomotor impairment or altered exploratory drive in tMCAO animals (**Figure** **2**). Analysis of total distance traveled (Figure 2A) showed no significant main effect of Group (*F* (1, 33.8) = 3.89, *p* = 0.057). Across testing sessions, tMCAO animals consistently exhibited equal or greater locomotor activity compared to Sham controls: Day 3 (Sham: 4.73 ± 3.03 m vs. tMCAO: 6.43 ± 2.65 m), Day 4 (Sham: 4.69 ± 3.48 m vs. tMCAO: 5.82 ± 3.51 m), Day 5 (Sham: 3.27 ± 2.18 m vs. tMCAO: 6.78 ± 2.45 m), and Day 29 (Sham: 5.26 ± 3.92 m vs. tMCAO: 7.00 ± 2.36 m). Post hoc analysis revealed a marginal group difference when averaged across all timepoints (*p* = 0.052), with the direction of effect inconsistent with motor impairment. There was no significant main effect of day (*F* (1, 67.9) = 1.45, *p* = 0.233) or Group × Day interaction (*F* (1, 67.9) = 0.099, *p* = 0.754), indicating stable activity levels throughout the observation period.

Vertical exploratory activity, quantified as rearing frequency (Figure 2B), showed no significant main effect of Group (*F* (1, 49.2) = 0.073, *p* = 0.788). However, a marginally significant Group × Day interaction emerged (*F* (1, 69.0) = 3.89, *p* = 0.052), driven primarily by divergent trajectories at Day 29 where tMCAO animals exhibited numerically elevated rearing compared to Sham controls (tMCAO: 15.6 ± 7.1 vs. Sham: 8.9 ± 5.6 rearings). This pattern suggests enhanced rather than diminished exploratory motivation in stroke animals at the late observation timepoint. The consistently elevated locomotor activity and increased rearing observed in tMCAO animals across all testing days may reflect post‐stroke hyperactivity and reduced anxiety‐like behavior, both of which have been reported following striatal and frontal lesions in rodents.

Assessment of anxiety‐related behavior through time allocation between arena zones revealed no significant group differences (Figure 2C). Time spent in the center zone, which inversely reflects anxiety‐like behavior, did not differ between groups (*F* (1, 89.0) = 1.91, *p* = 0.170). The Group × Day interaction was also nonsignificant (*F* (1, 89.0) = 0.031, *p* = 0.860), indicating stable emotional behavior across testing occasions in both experimental groups.

### 3.7 Neuroscore Assessment

**FIGURE 3:**
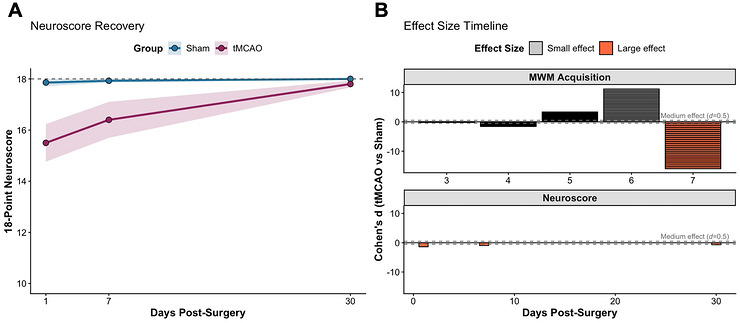
Temporal dissociation between sensorimotor and cognitive recovery. (A) Neuroscore recovery. Note that 18‐point composite neuroscore (mean ± 95% confidence interval) assessed on days 1, 7, and 30 post‐surgery in Sham (blue, *n* = 14) and tMCAO (magenta, *n* = 10) groups. Maximum score of 18 indicates normal function. (B) Effect size timeline. Cohen's *d* effect sizes comparing tMCAO versus Sham groups across recovery period for Morris water maze (MWM) acquisition (days 3–7, upper panel) and neuroscore (days 1–30, lower panel). Bars are color‐coded by magnitude: small effect (|*d*| < 0.5, gray), medium effect (0.5 ≤ |*d*| < 0.8, striped), and large effect (|*d*| ≥ 0.8, red). Dashed horizontal line at *d* = 0 indicates no group difference; reference line at *d* = ± 0.5 indicates threshold for medium effect. MWM, Morris water maze; tMCAO, transient middle cerebral artery occlusion.

Clinical neuroscore assessment using the 18‐point composite scale revealed clear group differences that evolved systematically over the 30‐day observation period (Figure 3A). Linear mixed‐effects analysis confirmed a significant Group × Day interaction (*F* (1, 46.0) = 16.36, *p* < 0.001), indicating that temporal recovery patterns differed between groups.

At Day 1 post‐surgery, tMCAO animals exhibited marked sensorimotor deficits relative to Sham controls (tMCAO: 15.5 ± 2.3 vs. Sham: 17.9 ± 0.5 points; mean difference: 2.4 points, *t* (22) = 3.37, *p* = 0.003). Only 10% (1/10) of tMCAO animals achieved the maximal score of 18 points at this acute timepoint, compared to 93% (13/14) of Sham animals. By Day 7, partial functional recovery was evident, although tMCAO animals remained impaired relative to controls (tMCAO: 16.4 ± 2.2 vs. Sham: 17.9 ± 0.3 points; mean difference: 1.5 points). The proportion of tMCAO animals achieving perfect scores increased to 50% (5/10) at this intermediate timepoint. At the final assessment on Day 30, near‐complete recovery had occurred, with minimal residual group differences (tMCAO: 17.8 ± 0.4 vs. Sham: 18.0 ± 0.0 points; mean difference: 0.2 points, *p* = 0.34). By this late timepoint, 80% (8/10) of tMCAO animals attained perfect scores, approaching the 100% rate observed in Sham animals (14/14). The gap between Day 7 and Day 30 assessment timepoints represents a limitation of the neuroscore data; intermediate assessments at days 14 or 21 would have better characterized the recovery trajectory and the timepoint at which functional equivalence between groups is achieved. This is acknowledged as a recommendation for future studies using this model.

### 3.8 Temporal Dissociation between Functional Domains

Neuroscore assessment detected substantial sensorimotor deficits at Day 1 that progressively resolved across the 30‐day observation period, conforming to a recovery trajectory consistent with that previously described for the 2‐h intraluminal filament tMCAO model in rats (Garcia et al. 1995; Wakayama et al. 2007). In stark contrast, MWM and Open field testing revealed no detectable spatial cognitive or complex motor impairments at any timepoint from Day 3 onward.

Effect size analysis quantified this temporal dissociation (Figure 3B). Neuroscore comparisons yielded large effect sizes at Day 1 (Cohen's *d* > 1.0), diminishing to medium effects by Day 7 (*d* = 0.7), and approaching negligible magnitudes by Day 30 (*d* < 0.2). Conversely, MWM acquisition comparisons across days 3–7 produced consistently small, non‐systematic effect sizes (|*d*| < 0.7) with no directional bias. Open field measures similarly yielded small effect sizes (|*d*| < 0.5) that, when significant, trended toward increased activity in tMCAO animals rather than impairment. The 95% confidence intervals around all MWM‐derived Cohen's *d* values were wide and crossed zero at every timepoint (acquisition range: −1.47–1.30; transfer trials: −0.80–1.16; reversal: −1.06–1.33), reflecting the limited precision inherent to this sample size and precluding firm conclusions about the true magnitude or direction of any group difference in spatial cognition. Sensitivity analysis indicated that, given the sample sizes employed (Sham *n* = 14, tMCAO *n* = 10), the study was powered to detect between‐group effect sizes of *d* ≥ 1.21 at 80% power (*α* = 0.05); the observed neuroscore effect at Day 1 (*d* = 1.53) confirms that effects of this magnitude were reliably detected when present.

## 4 Discussion

This post hoc exploratory analysis observed a dissociation between sensorimotor and spatial cognitive functional domains following tMCAO in rats. Acute sensorimotor deficits progressively resolved over 30 days, while spatial learning, short‐ and long‐term memory, cognitive flexibility, and open field locomotion appeared largely intact throughout the tested timeframe. These findings are consistent with the interpretation that the 2‐h transient occlusion model may produce injury sufficient to cause sensorimotor impairment while leaving spatial cognitive performance largely intact, with potential implications for the design and interpretation of preclinical stroke studies. It should be noted that the cognitive domains assessed were limited to spatial navigation, reference memory, and cognitive flexibility as measured by reversal learning. Other domains relevant to post‐stroke cognitive impairment in humans, including attention, working memory, and recognition memory, were not evaluated, and conclusions should not be extended beyond the specific domains tested.

The absence of MWM deficits in our tMCAO cohort aligns with Bouët et al. (2007), who reported intact spatial acquisition and probe trial performance in mice, though persistent sensorimotor deficits at 4 weeks were detected using more sensitive paradigms not captured by standard neurological scoring.

Similarly, Bingham et al. (2012) concluded that spatial memory deficits were minimal in a permanent electrocoagulation MCAO model despite apparent water maze impairments by conventional measures, attributing increased escape latencies and path lengths to sensorimotor dysfunction rather than cognitive impairment. Our study extends these observations in several respects: we employed a transient rather than permanent occlusion model; our 30‐day multi‐timepoint design confirms that the sensorimotor‐spatial cognitive dissociation persists across both acute and chronic recovery phases; we additionally demonstrated preserved cognitive flexibility through reversal learning; and open field analysis provided converging evidence that complex voluntary motor behaviors remain intact despite acute neurological deficits.

Roof et al. (2001) demonstrated that lesion location critically determines behavioral outcome in MCAO models, with permanent proximal MCAO producing combined cortical‐striatal injury and persistent MWM deficits, while cortical‐only distal MCAO showed sensorimotor recovery by 2 weeks and no water maze impairments. Our findings diverge from the former despite employing a similarly distributed cortical–striatal injury, and the critical distinction is likely occlusion permanence: 2‐h temporary occlusion with reperfusion may permit preservation of neural circuits sufficient to support spatial cognition even when striatal tissue is damaged. Methodological differences in water maze protocols may also contribute, as Roof et al. assessed performance at Day 7 with only six training trials, whereas our protocol began at Day 3 with four daily trials across five consecutive days, providing greater opportunity for compensatory strategy development.

Lapi et al. (2013) provide mechanistic context for our findings through direct in vivo visualization of pial microvascular remodeling in rats subjected to the same 2‐h MCAO model. They demonstrated near‐complete loss of perfused microvasculature in the ischemic core at 1‐h post‐reperfusion, followed by progressive formation of anastomotic arteriolar arcades and proliferation of penetrating arterioles that exceeded Sham values by Day 28. This reperfusion‐dependent vascular remodeling likely distinguishes transient from permanent occlusion models in ways that impact functional outcomes. The temporal mismatch between acute sensorimotor deficits, peaking at Day 1 when vascular damage is maximal, and preserved spatial cognitive performance from Day 3 onward, when vascular reorganization is already underway, is consistent with at least two interpretations that cannot be distinguished on the basis of the present data. First, reperfusion‐dependent vascular remodeling may preferentially rescue cognitive networks while striatal sensorimotor circuits remain more vulnerable to the acute ischemia‐reperfusion insult. Alternatively, and perhaps more parsimoniously, the territory affected by 2‐h intraluminal filament occlusion may simply not cause sufficient or appropriately located injury to disrupt the neural substrates of spatial cognition, a degree of structural damage that may require either longer occlusion durations or the more extensive cortical‐striatal injury associated with permanent proximal occlusion models. Both explanations are compatible with the observed dissociation, and future studies incorporating detailed lesion topography and targeted cognitive assessment would be needed to distinguish between them.

### 4.1 Limitations

Several limitations of the present study warrant consideration. First, our analysis was restricted to male Wistar rats, precluding generalization to female subjects or other rat strains. Sex differences in stroke pathophysiology and functional recovery are well‐documented, with female animals typically exhibiting smaller infarct volumes and enhanced recovery profiles relative to males. The exclusive use of male subjects limits the translational relevance of our findings to the substantial proportion of human stroke patients who are female. Second, behavioral assessment commenced on Day 3 post‐stroke, after the acute inflammatory cascade had been initiated. Earlier assessment during the hyperacute phase (24–48 h) might have revealed transient cognitive impairments that resolved prior to our first testing session. Histological assessment of infarct volume was available for 7 of 10 tMCAO animals, with the remainder allocated to molecular biological analysis. While infarct volumes confirmed successful stroke induction in all assessed animals, the absence of lesion topography data, particularly regarding hippocampal involvement and white matter injury, limits interpretation of the preserved spatial cognitive performance observed. In vivo neuroimaging or detailed post‐mortem histological mapping of lesion distribution would have substantially strengthened the interpretation of our behavioral findings. Third, the present analysis was not prospectively powered to detect cognitive outcomes, as it derives from an existing dataset collected for a different primary purpose. A classical post hoc power calculation is not informative in this context, as it is mathematically coupled to the observed p‐value and adds no independent information (Hoenig and Heisey 2001). As reported in the Results, the study was powered to detect effect sizes of *d* ≥ 1.21 at 80% power; medium‐sized cognitive effects (*d*  ~0.5) carried only approximately 21% power, meaning clinically relevant but moderate cognitive differences may have gone undetected. This uncertainty is reflected in the wide confidence intervals around all cognitive effect size estimates.

## 5 Conclusion

Within the testing window employed (days 3–30), 2‐h tMCAO in male Wistar rats produced acute sensorimotor deficits that resolved over 30 days, while spatial learning, memory consolidation, cognitive flexibility, and locomotion appeared intact throughout. These findings extend previous reports attributing conventional water maze impairments after experimental stroke to sensorimotor rather than cognitive dysfunction and raise the possibility that this model may not reliably replicate the cognitive consequences of human stroke within this testing paradigm. Future studies with dedicated prospective designs, histological verification, and broader cognitive batteries would be needed to confirm these observations. Behavioral test batteries may benefit from emphasizing sensorimotor endpoints, cognitive benefits of candidate therapeutics should be interpreted cautiously in the absence of baseline deficits, and alternative models may warrant consideration when post‐stroke cognitive dysfunction is the primary target. Future work should systematically compare outcomes across occlusion durations, reperfusion timings, and vessel occlusion sites. As a post hoc analysis of an existing dataset, this study additionally exemplifies the principle of reduction within the 3Rs framework for humane animal research.

## Author Contributions

**Michael Veldeman**: investigation, writing – original draft, methodology, visualization, data curation. **Mark Coburn**: supervision, project administration, conceptualization, funding acquisition, writing – review & editing, resources. **Anke Hoellig**: writing – review & editing, supervision, resources, project administration, conceptualization.

## Funding

This project was made possible by the generous funding of the German Research Foundation (Deutsche Forschungsgemeinschaft, DFG), Grant approval number: CO 799/ 9‐1.

## Ethics Statement

The research protocol and animal care procedures of this study were approved by the government agency for animal use and protection (AZ 84‐ 02.04.2013.A418, “Landesamt für Natur, Umwelt und Verbraucherschutz NRW,” Recklinghausen, Germany). All experiments were performed in accord‐ ance with the Guide for the Care and Use of Laboratory Animals (National Research Council [United States] and the Committee for the Update of the Guide for the Care and Use of Laboratory Animals, eighth Edition, 2011).

## Consent

The authors have nothing to report.

## Conflicts of Interest

Anke Hoellig lectured for Air Liquid Santé International. Mark Coburn consulted and lectured for Baxter Healthcare and Air Liquide. Michael Veldeman and J.L. declare no conflicts of interest.

## Data Availability

The raw data of this analysis can be made available by the authors to any qualified researcher upon reasonable request.
